# Investigation on a Novel Reinforcement Method of Grouting Sleeve Connection Considering the Absence of Reserved Reinforcing Bars in the Transition Layer

**DOI:** 10.3390/ma17235961

**Published:** 2024-12-05

**Authors:** Sheng Gu, Jun Yang, Saifeng Shen, Xing Li

**Affiliations:** 1 Kunshan Construction Engineering Quality Testing Center Co., Ltd., Kunshan 215337, China; s759654257@163.com; 2 Jiangsu Key Laboratory of Structure Engineering, Suzhou University of Science and Technology, Suzhou 215011, China; yangjun@usts.edu.cn (J.Y.); 230189492@seu.edu.cn (X.L.); 3 Key Laboratory of Concrete and Prestressed Concrete Structures of the Ministry of Education, Southeast University, Nanjing 211189, China; 4 School of Civil Engineering, Southeast University, Nanjing 211189, China

**Keywords:** reinforcement method, grouting sleeve connection, defect, reserved reinforcing bar, angle steel connector

## Abstract

In practical engineering, due to quality inspections of connections between prefabricated components and construction errors, reserved reinforcing bars in the transition layer may be partially insufficient or even completely absent. This defect significantly impacts the structural performance of sleeve connections, particularly under tensile or shear forces. This paper proposes a novel reinforcement method to address the connection issues caused by the absence of reserved reinforcing bars in the transition layer and verifies its feasibility through systematic experiments. To this end, this paper proposed a novel reinforcement method of grouting sleeve connection considering the absence of reserved bars in the transition layer, and 45 specimens with different reinforcement parameters were fabricated and tested under tension. Before verifying the reliability of the novel reinforcement method, nine specimens were fabricated and tested to verify the weldability of grouting sleeves and reinforcing bars. According to the test results, the fully grouted sleeves, including Grade 45 steel and Q345, showed good weldability with the HRB400 steel bars, while the ductile iron grouted sleeve showed poor weldability. When the single-sided welding length was greater than or equal to six times the diameter of the post-retrofitted connecting steel bar (*D*_2_), the primary failure mode observed in specimens utilizing the novel reinforcement method was the fracture of the prefabricated steel bar. The novel reinforcement method could be used to repair the defect of the grouting sleeve connection considering the absence of reserved reinforcing bars in the transition layer. When the single-sided welding length was 4*D*_2_, with a relative protective layer thickness of 2*D*_2_, and using C60 grade reinforcement material, this combination of conditions represented the critical condition to avoid weld failure between the grouting sleeve and the post-retrofitted connecting steel bars. In practical reinforcement projects, it is suggested that the single-sided welding length should be 5*D*_2_, the relative protective layer thickness should be 3*D*_2_, and the reinforcement material strength should be C60.

## 1. Introduction

In recent years, with the shortage of labor and the advancement of industrialization in the construction industry, precast concrete structures have been widely investigated and applied. Precast concrete structures have become an important trend in the current construction industry, bringing significant advantages in improving construction quality, saving costs, and shortening construction periods. They also provide an effective solution for addressing labor shortages and other related issues [1,2,3]. A precast concrete structure is a construction method where components like walls, stairs, and slabs are factory-produced, transported to the construction site for hoisting, and assembled through effective connections. Therefore, the quality of connections between components is crucial, as it directly impacts the overall stability of the structures [4,5]. There are various methods for component connections in precast concrete structures, and the sleeve grouting connection method is widely used due to its convenient construction process and good load-bearing performance. This connection method involves embedding sleeves within precast concrete components, inserting reinforcing bars into the sleeves, and finally filling them with grouting materials to ensure effective load transfer between adjacent components [6,7].

In practical engineering, grouting sleeve connections often exhibit defects that can compromise their performance. Previous studies have examined the mechanical properties of grouting sleeves with various defects. Guo et al. investigated the connection performance of fully grouted sleeve connectors with different grouting defects. They observed that a reduction in the effective anchor length of the connecting reinforcing bars significantly led to a deterioration in connection properties [8]. Kahama et al. investigated the impact of grouting defects on the mechanical properties of a full-grouted sleeve connector through numerical simulation. They discovered that reducing the sleeve diameter could enhance the connection performance. In the weakest configuration, the minimum anchorage of the bar needed for adequate bonding was found to be eight times the diameter of the bar [9]. Liu et al. conducted cyclic loading tests on ninety-nine specimens of half-grouted sleeves with defects. They analyzed the stress transfer path of the defective grout material and its restraining effect on the sleeve. Their findings revealed that a concentrated and laterally distributed defect resulted in more severe deterioration compared to a uniformly dispersed defect [10]. Qu et al. performed a uniaxial tensile test on 33 specimens with grouting defects to investigate the mechanical properties of these defects. They determined that the specimen remained in a safe state when the anchorage length of the rebar exceeded six times the diameter of the connecting rebar [11]. Therefore, it can be seen that grouting defects could significantly impact the connection performance. The main factors affecting the connection performance of the grouting sleeve are the effective anchorage length of the connecting reinforcing bars and the distribution of grouting defects.

Merely studying the impact of defects on connections from the material perspective is not sufficient. Hence, scholars have extended their research to examine how grouting sleeve connection defects influence the overall structural integrity. For instance, Xiao et al. investigated the effects of sleeve grouting defects on the seismic performance of precast concrete shear walls, discovering that such defects notably influenced wall behavior when the spliced bar was under tension [12]. Yan et al. designed and established three finite element models of shear walls with full sleeve grouting, a fully defective sleeve, and a partially defective sleeve, and found that defects have an impact on the structural load-bearing capacity and failure process [13]. Furthermore, Yang et al. investigated the seismic behaviors of precast concrete shear walls with sleeve grouting defects and found that the grouting defects caused the weakening of the stiffness, ductility, and energy dissipation of the precast concrete shear walls [14]. Cao et al. carried out quasi-static tests on seven precast reinforced concrete shear walls with varying grouting defects and revealed that grouting deficiencies had minimal impact on initial stiffness but significantly reduced later stiffness, bearing capacity, and energy dissipation [15]. These collective studies confirm that grouting sleeve connection defects exert a substantial influence on the load-bearing capacity of structures, thereby compromising their seismic resistance.

In order to repair the defects in the grouting sleeve connection during the construction process, some scholars have proposed reinforcement methods for different defects. Zheng et al. repaired sleeve connections by refilling grout material to the sleeve enclosing the insufficient grout material, and they investigated the mechanical performance of defective and repaired grouted sleeve connections under uniaxial and cyclic loadings. The test results showed that the repaired sleeve connections showed similar mechanical performance to the fully grouted sleeve connection except for deformation [16]. Li et al. used a simple grout injection technique to repair two-story precast concrete frame structures having grout sleeve connections in their columns and found that the simple grout injection technique could well repair the grouting defect in the columns [17]. Xie et al. recommended a quantitative detection and repair method as well as associated endoscopic equipment for the defect detection and repair of half-grouted sleeve connections, and they validated their detectability and repairability through laboratory tests and fieldwork in real engineering practice [18]. Xie et al. also used this method to repair precast concrete (PC) columns with grouting defects. The feasibility and reliability of the repair method was validated by tests under cyclic loads, and the test results showed that this repair method almost eliminated the negative influences of insufficient grouting [19]. It can be seen that current reinforcement and repair methods are mostly developed for grouting defects, while there is little research on reinforcement methods for defects with insufficient anchorage length of connecting steel bars.

**Figure 1:**
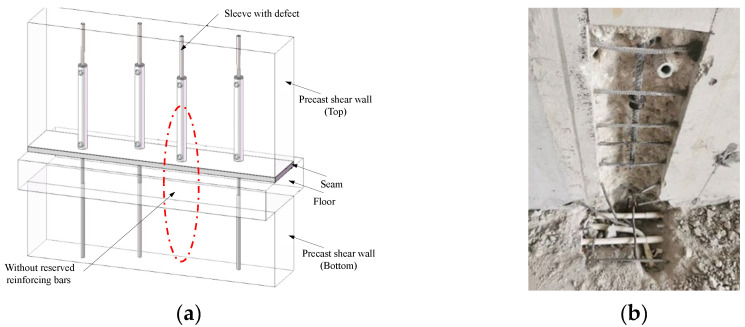
Component with insufficient reserved bars. (**a**) Schematic diagram. (**b**) Inspection diagram of a certain project site.

During the construction of precast concrete structures, the reserved reinforcing bars at the top of the lower prefabricated components need to be inserted into the sleeves reserved at the bottom of the upper prefabricated components and then grouted for a compact connection. However, as shown in Figure 1, with the development of grouting sleeve connection quality testing technology and the implementation of inspection work, it might be found that there are cases where the length of the reserved bars in the transition layer is insufficient, partially unreserved, or even entirely unreserved. This is because the connection between the upper section’s reinforcing bars and the grouting sleeves is pre-fabricated in the factory, where it is easier to ensure construction quality. However, the connection of the lower section’s reinforcing bars to the grouting sleeves during on-site construction often encounters defects due to factors such as precision in processing and worker operation. These defects can significantly impact the structural safety. In such situations, even if the sleeve is fully grouted, it cannot effectively establish a reliable connection. Therefore, reinforcing the insufficiently reserved reinforcing bars in the transition layer of precast concrete structures is deemed essential.

**Figure 2:**
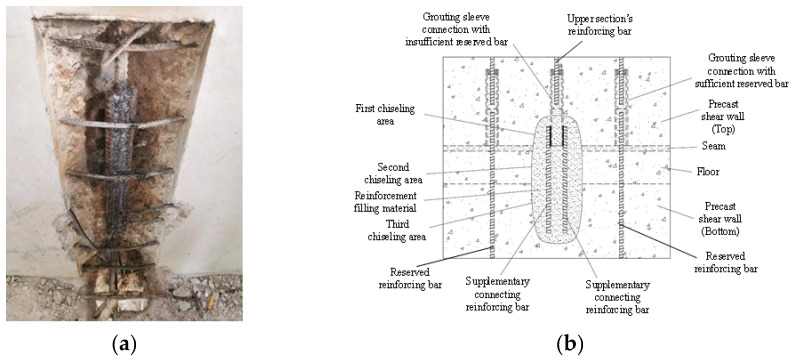
Reinforcement method. (**a**) Conventional reinforcement method. (**b**) Novel reinforcement method.

Regarding the deficiencies mentioned above, the current conventional reinforcement method used is shown in Figure 2a. This method requires removing the concrete around the grouting sleeve with insufficient reserved bars and extracting the grouting sleeve. Additionally, the lower reinforcement steel and upper connecting steel require a welding connection with two supplementary connecting reinforcing bars. This method demands a large welding length and causes significant damage to the concrete structure in the damaged area. To this end, this paper proposes a novel reinforcement method of grouting sleeve connection considering the absence of reserved bars in the transition layer, as shown in Figure 2b. Even in the case of grouting sleeves lacking reserved reinforcing bars, when they are fully grouted, their connection with the upper section’s reinforcing bars typically remains reliable. This method aims to fully utilize the residual value of grouting sleeve connections that fail to connect with the lower section’s reinforcing bars (reserved reinforcing bars). A first chiseling area was set around the grouting sleeve connection with insufficient reserved bars. The first chiseling area corresponded to a second chiseling area and a third chiseling area on the floor and the precast shear wall (bottom), respectively. The surface of the grouting sleeve connection with insufficient reserved bars in the first chiseling area was equipped with two supplementary connecting reinforcing bars. One end of the supplementary connecting reinforcing bars passed through the second chiseling area to the third chiseling area. The first chiseling area, the second chiseling area, and the third chiseling area were connected and filled with structural reinforcement filling material. The supplementary connecting reinforcing bars were welded and fixed on both sides of the outer wall of the prefabricated shear wall along the length direction of the grouting sleeves to ensure uniform and reliable force transmission. The sum of the cross-sectional areas of the two supplementary connecting reinforcing bars was greater than or equal to the cross-sectional area of the upper connecting steel bars, meeting the principle of equal section conversion for steel bar force transmission. To investigate the performance of this reinforcement method, 45 specimens with different reinforcement parameters were fabricated and tested under tension. Before verifying the reliability of the novel reinforcement method, nine specimens were fabricated and tested to verify the weldability of grouting sleeves and reinforcing bars.

## 2. Description of Experiments

### 2.1. Specimen Design

#### 2.1.1. Weldability of Grouting Sleeves and Reinforcing Bars

**Figure 3:**
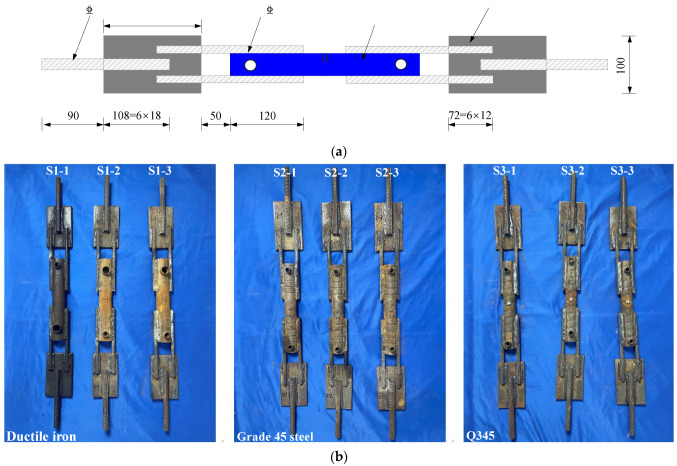
Specimens for the weldability test of grouting sleeves and reinforcing bars (unit: mm). (**a**) Dimensions and steel reinforcements of the specimens. (**b**) Specimens.

Currently, there are various materials used for grouting sleeves available on the market, such as ductile iron, Grade 45 steel, and Q345. Most grouting sleeves and reinforcing bars are fabricated from different materials, and the weldability between grouting sleeves and reinforcing bars should be verified. In addition, there are multiple manufacturing processes for grouting sleeves. For instance, in the case of sleeves produced by rolling, the threads on the outer surface of the sleeve are relatively deep, making it challenging to achieve a tight fit when welding with the reinforcing bars. Therefore, it is necessary to verify the weldability of grouting sleeves and reinforcing bars to ensure the reliability of the novel reinforcement method. This experiment selected three types of fully grouted sleeves, ductile iron (S1-1, S1-2, S1-3), Grade 45 steel (S2-1, S2-2, S2-3), and Q345 (S3-1, S3-2, S3-3), for the fabrication of the test specimens. The specimens S1-1, S1-2, and S1-3, as well as S2-1, S2-2, and S2-3 and S3-1, S3-2, and S3-3, were indeed identical in terms of their design and material properties. The purpose of these specimens was to assess the repeatability and reliability of the experimental results. Since the original connecting reinforcing bars inside the test sleeves had a diameter of 16 mm, supplementary connecting reinforcing bars with a diameter of 12 mm were chosen. The test specimens were designed as shown in Figure 3. A total of nine test specimens were fabricated, with three specimens of the same dimensions designed for each type of grouting sleeve, as shown in Figure 3b.

#### 2.1.2. Proposed Reinforcement Technique

This section presents the details of the novel reinforcement method for the welded joint between the retrofitted connecting steel bars and the sleeves. In practical engineering applications, after the one-sided welding of the retrofitted connecting steel bars to the sleeve is completed, it is necessary to fill the reinforcement material around the weld. Once the filling is completed, the load-bearing capacity of the joint relies on both the weld strength between the steel bars and the grouted sleeve and adhesive strength of the reinforcement material around the weld. Therefore, this experimental study included three aspects, namely, the one-sided lap welding length between the steel bars and the sleeves, the relative protective layer thickness, and the strength of the reinforcement filling materials. Therefore, this study designed 15 groups of specimens, with each group containing three identical specimens, as shown in Table 1.

**Figure 4:**
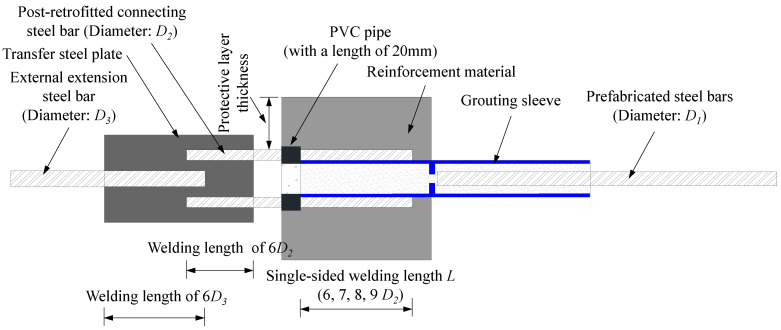
Specimen for the novel reinforcement method.

The connection between the grouted sleeve and the steel bar on the prefabricated side was realized by grouting materials. On both sides along the diameter direction of the post-retrofitted connecting steel, single-sided lap welding was used to weld two post-retrofitted connecting steel bars to the grouted sleeve. To ensure that both ends of the specimens were clamped at a single point during the loading process, the other end of the two post-retrofitted connecting steel bars was fixed to the transfer steel plate by double-sided lap welding. An external extension steel bar was welded onto the transfer steel plate. This ensured the uniform transmission of tension to the two post-retrofitted connecting steel bars. To investigate the influence of the one-sided lap welding length between the steel bars and the sleeves, welding lengths of 6*D*_2_, 7*D*_2_, 8*D*_2_, or 9*D*_2_ (where *D*_2_ was the diameter of the post-retrofitted connecting steel bar) were selected to fabricate the specimens. The external extension steel bar and the transfer steel plate were fixed by double-sided lap welding, with a welding length of 6*D*_3_ (where *D*_3_ was the diameter of the external extension steel bar). The diameters of the prefabricated steel bars inside the sleeves were 14 mm, 16 mm, and 18 mm for the different specimens. According to the principle of equivalent cross-section of steel bars, the corresponding diameters of the post-retrofitted connecting steel bars on both sides of the sleeves were 10 mm, 12 mm, and 14 mm, and the diameters of the external extension steel bars were 16 mm, 18 mm, and 20 mm, respectively. The details of the specimens are shown in Figure 4.

**Figure 5:**
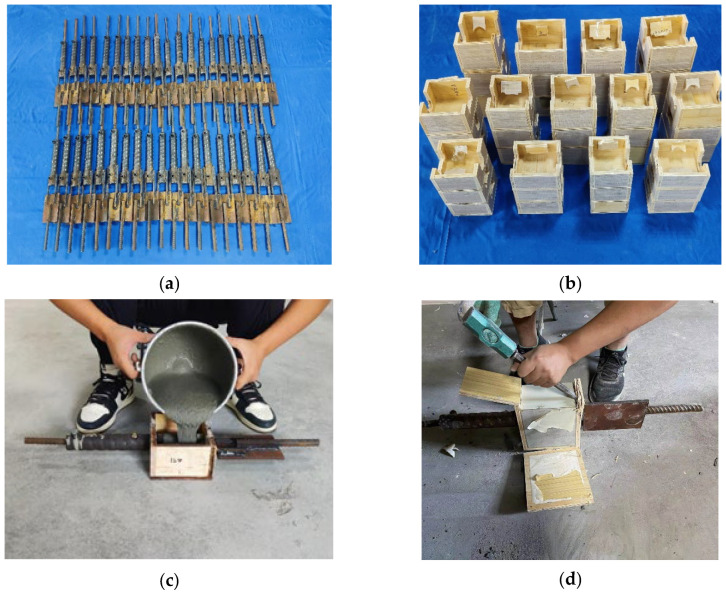
Preparation process of the specimens. (**a**) Sleeve welding. (**b**) Wooden mold making. (**c**) Injecting reinforcement materials into the mold. (**d**) Demolding.

The specimen preparation process, as shown in Figure 5, began with single-sided lap welding along the radial direction at the bottom installation end of the sleeve. Two post-connection reinforcing bars were welded to the sleeve and further connected to a force transfer steel plate. Subsequently, an external extension bar was welded to the steel plate. After completing the welding and allowing it to cool, sealing rings were installed at both the prefabricated and installation ends of the sleeve. The center hole of the sealing ring at the installation end was sealed with tape to prevent grout leakage. Next, the connecting steel bar inside the sleeve was inserted through the center hole of the sealing ring at the prefabricated end of the sleeve, and the completed specimen was placed into a wooden mold. The interface between the sleeve and the mold was sealed with clay to prevent grout leakage during the grouting process. To avoid local compression during loading, the two post-connection reinforcing bars were wrapped in 20-mm-long PVC isolation sleeves, and clay was packed between the sleeves and the bars to prevent grout intrusion, ensuring accurate experimental results. During grouting, the sleeve was positioned horizontally, with PVC extension pipes vertically inserted into both the grouting inlet and outlet. Grouting was performed from the outlet to prevent grout from entering the mold. The rise of grout in the PVC pipe at the inlet was monitored, and grouting was stopped once grout slowly ascended in this pipe. After grouting, the specimen was left undisturbed for approximately one hour until the grout achieved its initial set. The PVC pipes at the inlet and outlet were then removed, and reinforcement material was poured into the mold. After a 28-day curing period, the mold was removed, and the specimen was subjected to uniaxial tensile testing.

### 2.2. Material Properties

This experiment used commercial fully grouted sleeves, and their mechanical properties are shown in Table 2. HRB 400 steel bars were adopted in all specimens. The properties of steel bars were tested in accordance with the Chinese code for Design of Concrete Structures (GB 50010-2010) [20], and the results are shown in Table 3. According to the Technical Specification for Application of Cement-based Grouting Materials (GB/T 50448-2015) [21], three standard 40 mm × 40 mm × 160 mm prismatic specimens were prepared for each grouting material. After curing for 28 days, compressive strength tests were conducted on them, and the test results are shown in Table 4.

### 2.3. Test Setup and Load Protocol

#### 2.3.1. Test of Weldability of Grouting Sleeves and Reinforcing Bars

**Figure 6:**
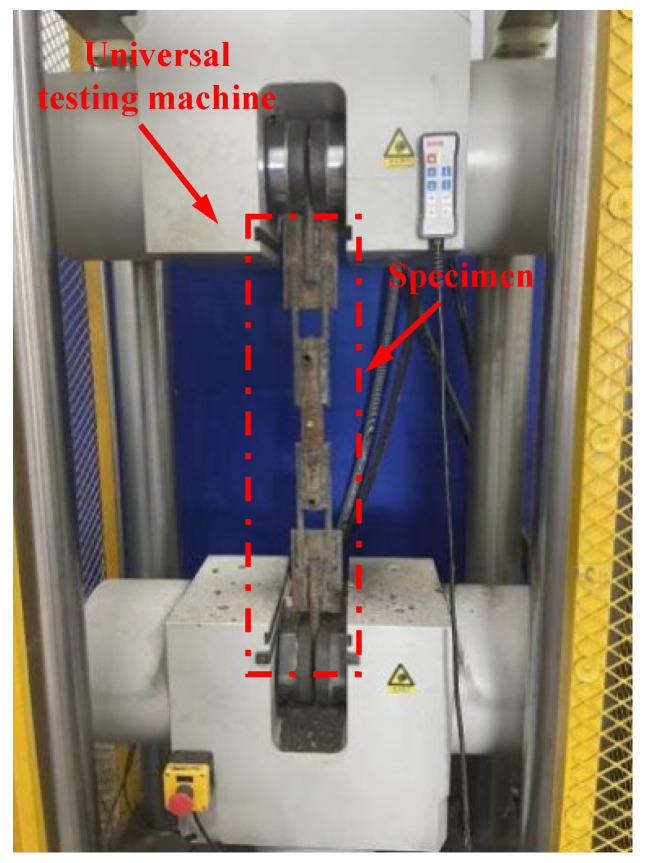
Test setup of the weldability test.

The tensile test was conducted using the universal testing machine at the Kunshan Construct Engineering Quality Testing Center, as shown in Figure 6. During the tests, the specimens were clamped at both ends with the two external extension steel bars. In this experiment, the distance between the fixtures of the testing machine was set at 730 mm. According to the Standard for Test Methods of Welded Joints of Steel Bars (JGJ/T27-2014) [22], the tensile process should be continuous and smooth until the specimen is pulled to fracture (or experiences constriction). The tensile rate was set as 36.5 mm/min ($V_{0}=0.05\times 730=36.5\mathrm{mm}/\min$).

#### 2.3.2. Test of Proposed Reinforcement Technique

This experiment employed the universal testing machine for static tensile testing. The loading ratio was referenced from the Technical Specification for Steel Bar Connections (JGJ107-2010) [23], and the tensile rate was set as 25.5 mm/min.

## 3. Experimental Results and Analysis

### 3.1. Weldability of Grouting Sleeves and Reinforcing Bars

To verify the weldability between the grouting sleeve and the steel bars, tensile tests were conducted using single-sided lap welding. While the test setup may resemble conventional strength tests, its primary purpose was to evaluate the reliability of weld joints under conditions relevant to the proposed novel reinforcement method. The inclusion of the prefabricated steel bar and its concrete anchorage was necessary to simulate realistic loading conditions and ensure proper clamping of the specimen in the testing machine. This design allowed for an accurate representation of the force transmission and failure behavior of the connection under tensile load. It is important to note that the prefabricated steel bar itself was not the focus of the evaluation but rather a critical component to facilitate the testing of the welded joint and its surrounding elements. This setup ensured that the experimental results are reflective of practical engineering applications.

**Figure 7:**
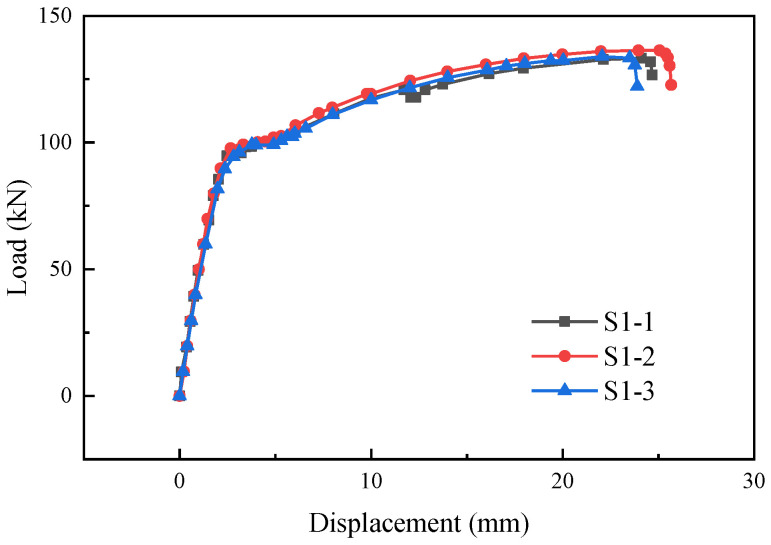
Load-displacement curves of specimens S1-1, S1-2, and S1-3.

**Figure 8:**
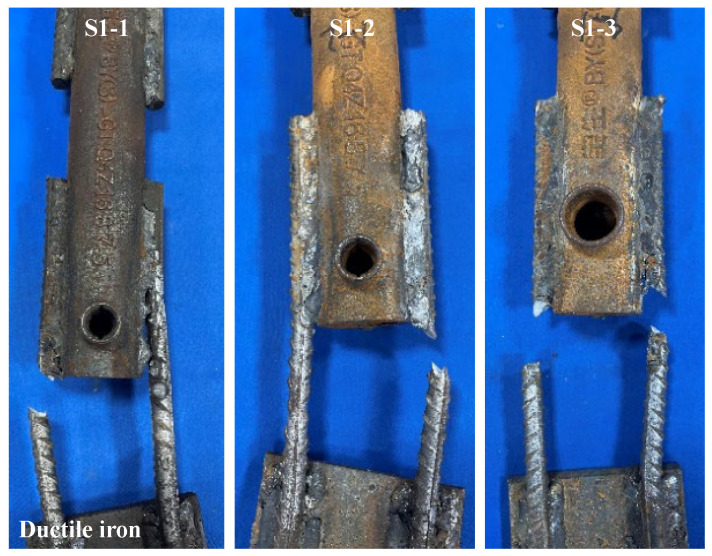
Failure modes of specimens S1-1, S1-2, and S1-3.

The load-displacement curves of specimens S1-1, S1-2, and S1-3 are shown in Figure 7. The load-displacement curves of the specimens were generally consistent with the load-displacement curves of ordinary steel bars under tension, including the elastic stage, yielding stage, strengthening stage, and necking stage. For specimen S1-1, after entering the strengthening stage, the weld between one supplementary connecting reinforcing bar and the sleeve cracked, resulting in a brief drop in load. Then, another weld seam between the supplementary connecting reinforcing bar and the sleeve underwent contraction and subsequently fractured, with the fracture occurring in the vicinity of the weld heat-affected zone. Specimens S1-2 and S1-3 experienced fractures of the supplementary connecting steel bars during the tension process, with the fracture occurring in the vicinity of the weld heat-affected zone. The failure modes of the specimens are shown in Figure 8.

**Figure 9:**
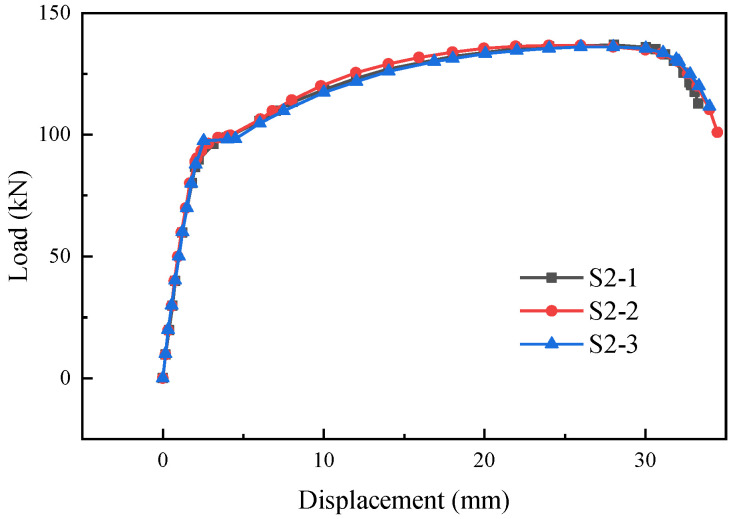
Load-displacement curves of specimens S2-1, S2-2, and S2-3.

**Figure 10:**
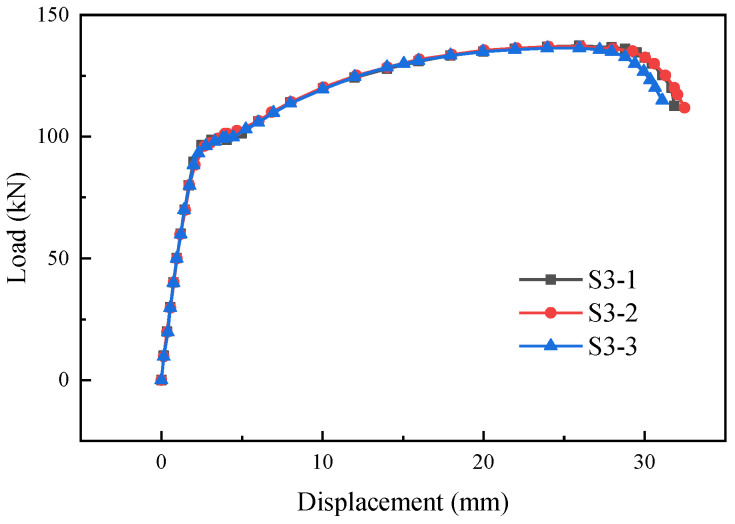
Load-displacement curves of specimens S3-1, S3-2, and S3-3.

**Figure 11:**
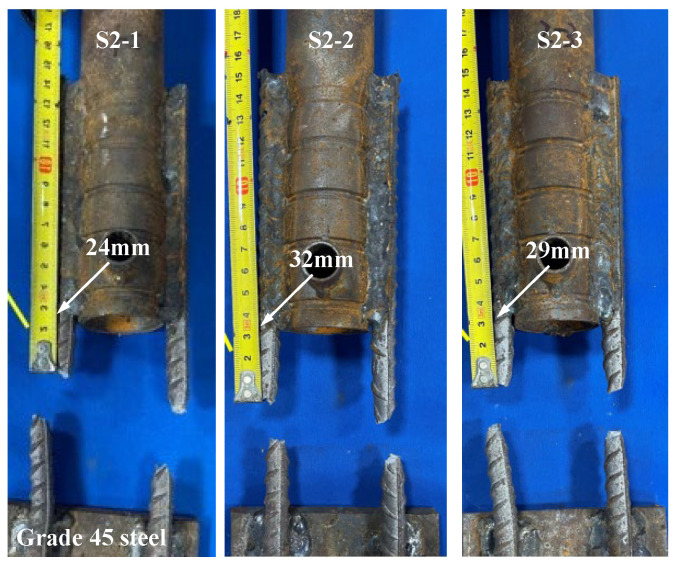
Failure modes of specimens S2-1, S2-2, and S2-3.

**Figure 12:**
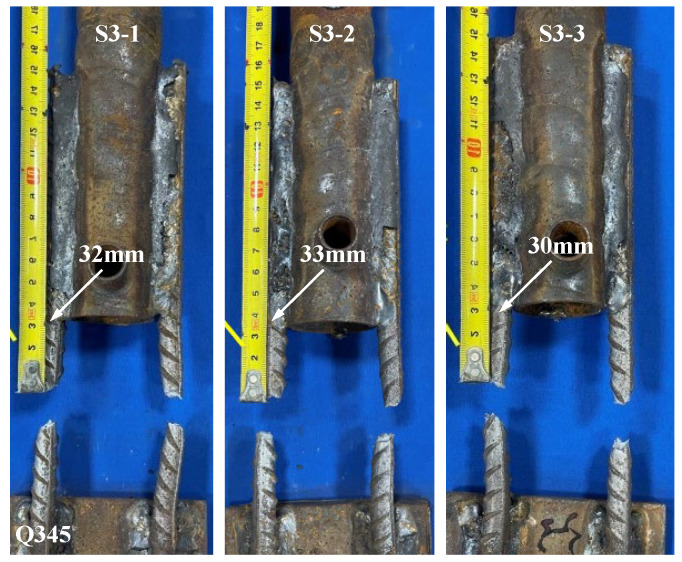
Failure modes of specimens S3-1, S3-2, and S3-3.

Figure 9 and Figure 10 show the load-displacement curves of specimens S2 and S3, respectively. The load-displacement curves of specimens S2 and S3 were essentially similar. In the initial stage, both ends of the specimens were subjected to force simultaneously with two supplementary connecting steel bars. As the load continued to increase, one of the supplementary connecting steel bars experienced a fracture. The loading process continued, and the other supplementary connecting steel bar also fractured rapidly. The failure of the specimens occurred within the predetermined failure zone, with the fracture location approximately 24 mm–33 mm from the weld seam, as shown in Figure 11 and Figure 12.

The tensile tests revealed three primary failure modes in the specimens: (1) Fracture of the supplementary connecting steel bar occurred in the weld heat-affected zone. (2) Fracture of the supplementary connecting steel bar occurred within the predetermined failure zone. (3) Failure of the weld joint occurred between the supplementary connecting steel bar and the grouting sleeve. The characteristic values of the tensile test for the specimens are shown in Table 5. The tensile strength of specimens S1-1, S1-2, and S1-3 was higher than the standard value of the steel reinforcements, but the failure zones of the specimens did not meet the regulatory requirements. For specimen S1-1, one of the supplementary connecting steel bars fractured in the weld heat-affected zone, and the weld joint suffered damage. For specimens S1-2 and S1-3, the supplementary connecting steel bars fractured in the weld heat-affected zone. According to the relevant regulations, the welded joints of specimens S1-1, S1-2, and S1-3 showed poor weldability. For specimens S2 and S3, the supplementary connecting steel bars of the specimens all fractured within the predetermined failure zone. They did not break within the heat-affected zone, and the steel bars exhibited ductile fracture. At the same time, the ultimate tensile strength of the specimens during the tensile process was greater than the ultimate tensile strength standard value of the steel reinforcements. According to the relevant regulations, the welded joints of specimens S2 and S3 showed good weldability.

### 3.2. Novel Reinforcement Method of the Grouting Sleeve Connection

**Figure 13:**
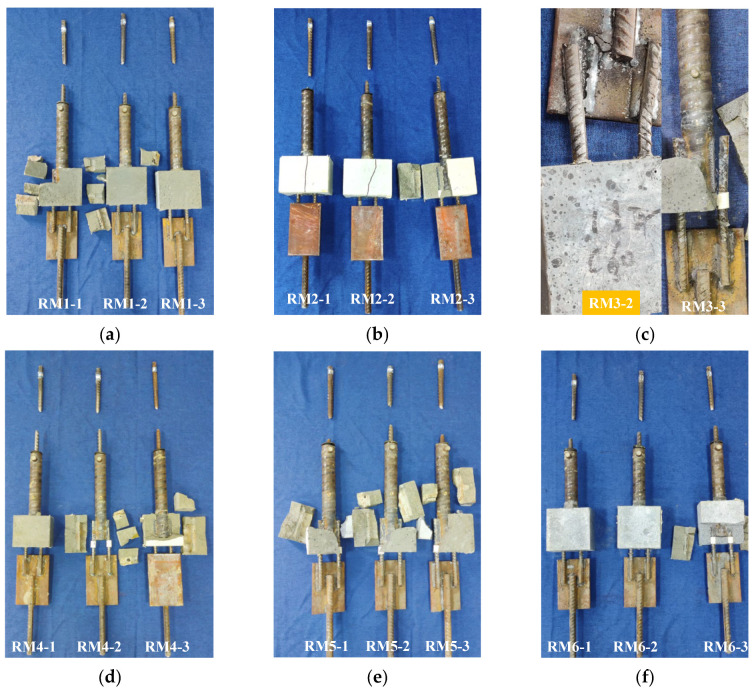

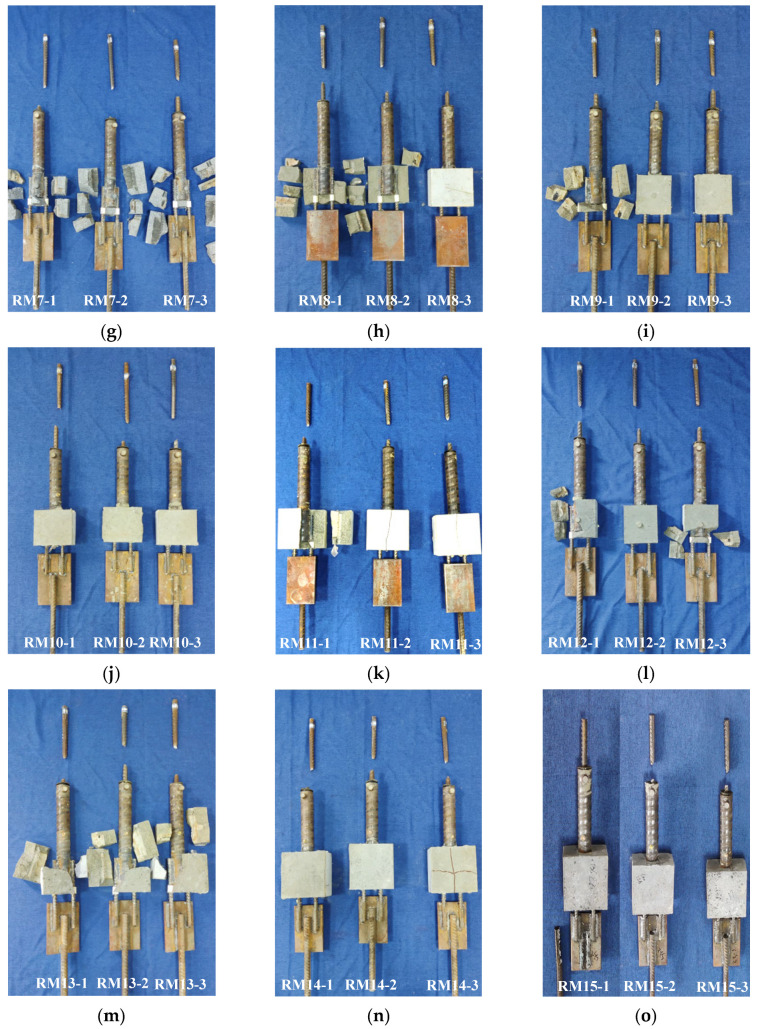
Failure modes of the specimens. (**a**) RM1. (**b**) RM2. (**c**) RM3. (**d**) RM4. (**e**) RM5. (**f**) RM6. (**g**) RM7. (**h**) RM8. (**i**) RM9. (**j**) RM10. (**k**) RM11. (**l**) RM12. (**m**) RM13. (**n**) RM14. (**o**) RM15.

There were three failure modes of the specimens: (1) Weld failure occurred at the transfer steel plate (failure type: M), which included failure at the weld joint between the external extension steel bar and the transfer steel plate as well as failure at the weld joint between the post-retrofitted connecting steel bar and the connecting steel plate. (2) Fracture failure of the prefabricated steel bar occurred (failure type: P). (3) Weld failure occurred between the grouting sleeve and the post-retrofitted connecting steel bars (failure type: N). Figure 13 shows the failure modes of the specimens. The results of the tensile tests on the specimens are presented in Table 6.

**Figure 14:**
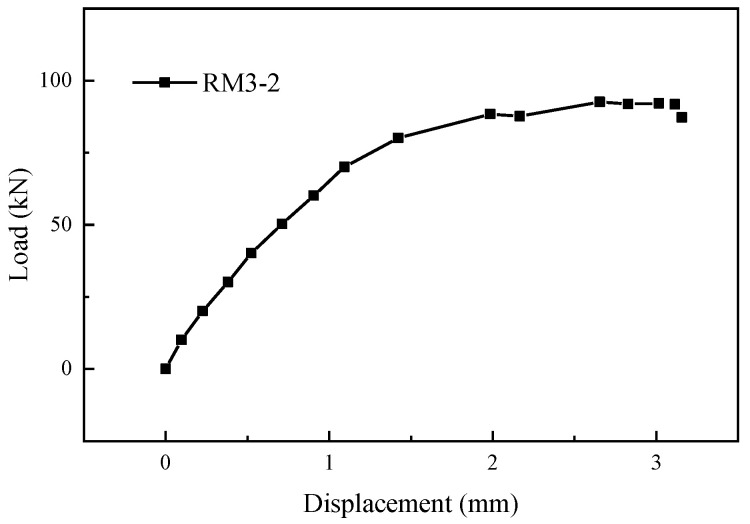
Load-displacement curve of specimen RM3-2.

**Figure 15:**
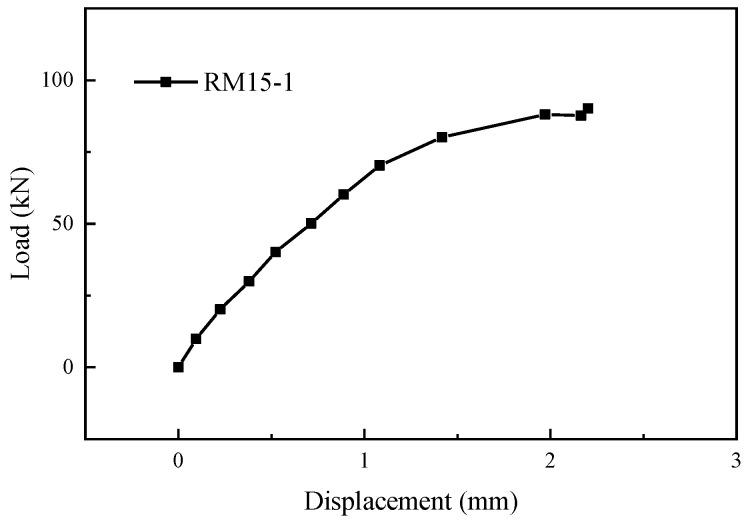
Load-displacement curve of specimen RM15-1.

As shown in Figure 13c,o, Specimen RM3-2 experienced a failure at the weld joint between one of the post-retrofitted connecting steel bars and the transfer steel plate, and Specimen RM15-1 suffered a failure at the weld joint between the external extension steel bar and the transfer steel plate. Figure 14 and Figure 15 show the load-displacement curves of specimens RM3-2 and RM15-1, respectively. In the elastic phase, the load-displacement curves showed a linear increase. During the loading process, a slight sound could be heard coming from the weld joint. When the tensile force approached the yield point of the specimens, a sudden failure occurred at the weld joint. The entire weld joint between the transfer steel plate and the post-retrofitted connecting steel bars was pulled apart. The ultimate load for specimens RM3-2 and RM15-1 were 92.86 kN and 90.13 kN, respectively, which was below the ultimate tensile load standard value of 108.59 kN for the HRB400 steel bar. The main reason for this discrepancy was attributed to inadequate welding by the workers.

**Figure 16:**
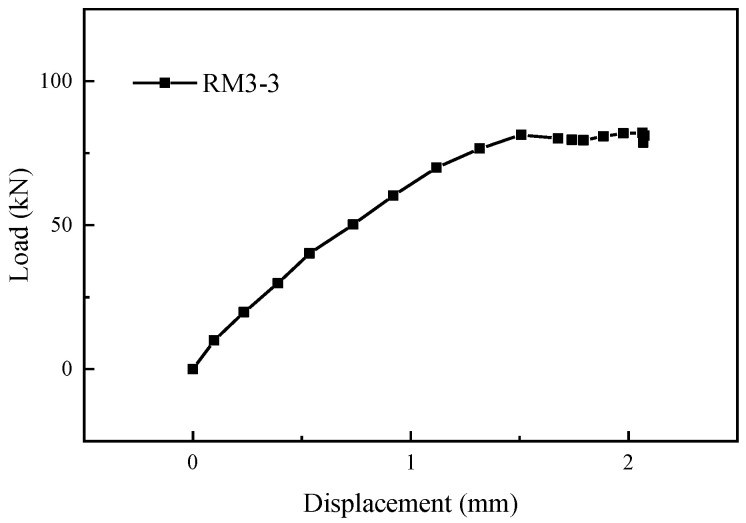
Load-displacement curve of specimen RM3-3.

As shown in Figure 13c, specimen RM3-3 experienced weld failure between the grouting sleeve and the post-retrofitted connecting steel bars. Figure 16 shows the load-displacement curves of specimen RM3-3. It can be seen that in the initial stage of the tensile test, the load-displacement curve exhibited a linear increase. When the force reached 84.13 kN, a failure occurred at the weld joint. At the moment of failure, the grouting material around the weld joint broke apart.

**Figure 17:**
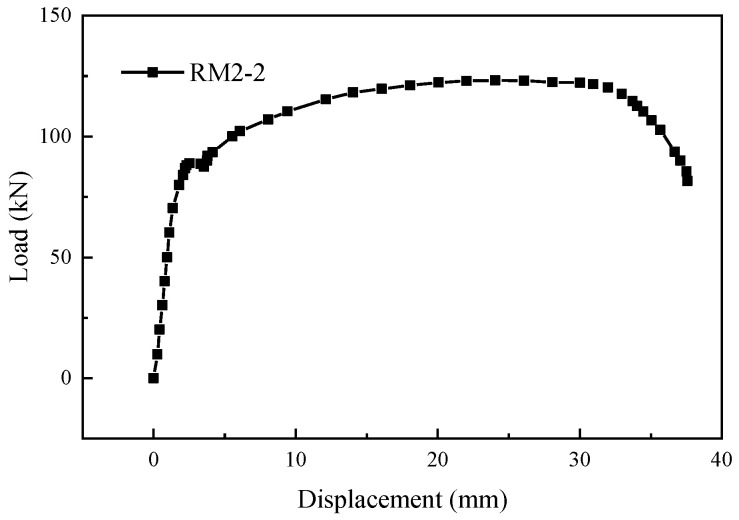
Typical load-displacement curve.

As shown in Figure 13, except for specimens RM3-2, RM-3-3, and RM15-1, the other specimens exhibited fracture failure of the prefabricated steel bar. When the single-side welding length was larger than 6*D*_2_, and the grouting material strength was higher than C40, the specimens generally experienced fracture failure of the prefabricated steel bar. Throughout the entire tensile process, there were no significant changes observed in the load-displacement curves of the specimens. As shown in Figure 17, the typical load-displacement curve during the tensile test of the specimens matched the load-displacement curve of a reinforcement tensile test. Both curves exhibited the elastic stage, yield stage, strengthening stage, and necking stage. After entering the necking stage, the external extension steel bars suddenly fractured. Simultaneously, the grouting material at the connection point between the grouting sleeve and the post-retrofitted connecting steel bars was crushed, causing detachment of the grouting material around the weld joint. However, all specimens retained their weld joints intact.

According to Table 6, it is evident that the most significant factor affecting the experimental results was the single-sided welding length. When the welding length exceeded 6*D*_2_, specimens exhibited fracture failure of the prefabricated steel bar. Therefore, a single-sided welding length of 6*D*_2_, a relative protective layer thickness of 3*D*_2_, and reinforcement material using C40 grout might represent the critical combination of conditions for potential weld failure between the grouting sleeve and the post-retrofitted connecting steel bars. To validate this critical condition for the weld failure between the grouting sleeve and the post-retrofitted connecting steel bars, it is necessary to reduce the welding length between the post-retrofitted connecting steel bar and the sleeve. Meanwhile, it is necessary to adjust the protective layer thickness as well as the strength of the grout material. To this end, nine groups of specimens were designed and fabricated, as shown in Table 7.

**Figure 18:**
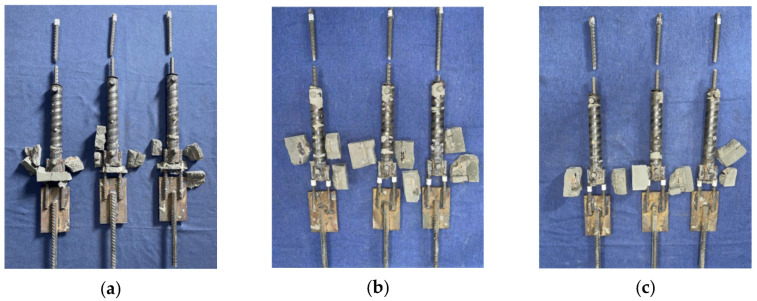

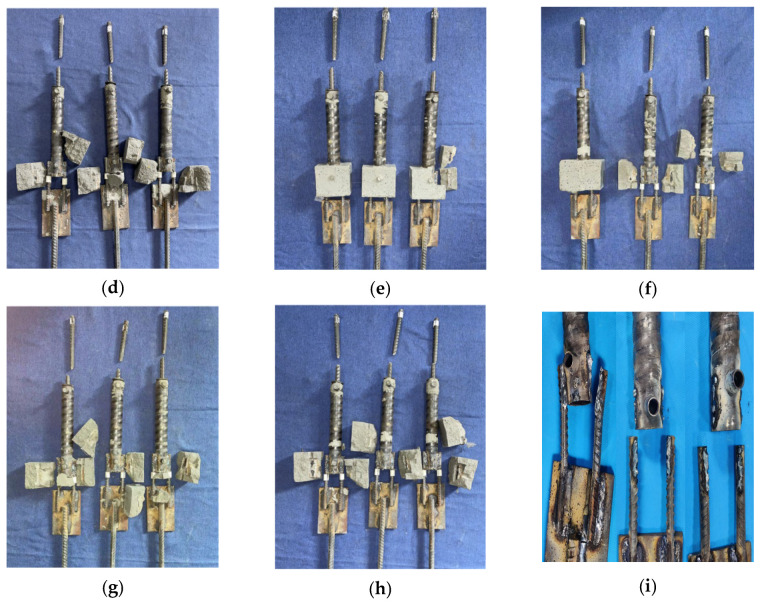
Failure modes of the supplementary specimens. (**a**) S-RM-1. (**b**) S-RM-2. (**c**) S-RM-3. (**d**) S-RM-4. (**e**) S-RM-5. (**f**) S-RM-6. (**g**) S-RM-7. (**h**) S-RM-8. (**i**) S-RM-9.

The test results of the supplementary specimens are shown in Table 8 and Figure 18. Except for specimens S-RM9-1, S-RM9-2, and S-RM9-3, the other specimens exhibited fracture failure of the prefabricated steel bar. During the tensile testing of the specimens, there was no apparent surface damage observed. At the moment when the prefabricated steel bars fractured, the grout material around the weld seam was shattered, but the weld seam between the sleeve and the post-retrofitted connecting steel bar remained intact. When the single-sided welding length between the post-retrofitted connecting steel bar and the sleeve was 4*D*_2_, and there was no grout material encapsulation around the weld seam, the specimens experienced weld failure between the grouting sleeve and the post-retrofitted connecting steel bars. However, the specimens with a single-side welding length of 4*D*_2_, and the C60 reinforcement material with a relative protective layer thickness of 2*D*_2_, exhibited fracture failure of the prefabricated steel bar. This indicated that the presence of grout material encapsulation around the weld seam provided a certain reinforcement. At the same time, this combination represented the critical condition for failure of type N. To ensure the quality of the reinforcement, it is suggested that the single-sided welding length should be no less than 5*D*_2_.

## 4. Conclusions

This paper presented a novel reinforcement method of grouting sleeve connection considering the absence of reserved bars in the transition layer. Through systematic experimental research, the weldability of the grouting sleeve and the reliability of the new reinforcement method were verified. The following conclusions were drawn.

1. The fully grouted sleeves, including Grade 45 steel and Q345, demonstrated good weldability with the HRB400 steel bars. It is important to note that weldability results are independent of whether the sleeves were fully grouted. Instead, weldability serves as a fundamental requirement for ensuring the feasibility of the proposed reinforcement method under conditions where reserved bars in the transition layer are absent or insufficient.
2. When the single-sided welding length was greater than or equal to 6*D*_2_, the primary failure mode observed in specimens utilizing the novel reinforcement method was the fracture of the prefabricated steel bar. Adjusting the strength of the grouting material and the thickness of the protective layer had little effect on the connection performance of the specimens.
3. The smaller the single-sided welding length, the more pronounced the reinforcing effect of the reinforcement material near the weld. When the single-sided welding length was 4*D*_2_, with a relative protective layer thickness of 2*D*_2_, and using C60 grade reinforcement material, this combination of conditions represented the critical condition to avoid weld failure between the grouting sleeve and the post-retrofitted connecting steel bars. In practical reinforcement projects, it is suggested that the single-sided welding length should be 5*D*_2_, the relative protective layer thickness should be 3*D*_2_, and the reinforcement material strength should be C60.

## Figures and Tables

**Table 1:** Parameters of the specimens.

Group No.	Diameter of Prefabricated Steel Bars *D*_1_ (mm)	Diameter of Post-Retrofitted Connecting Steel Bars *D*_2_ (mm)	Single-Sided Welding Length *L* (mm)	Protective Layer Thickness *D* (mm)	Strength of Reinforcement Material	Number of Specimens
RM1	16	12	6*D*_2_	3*D*_2_	C85	3
RM2	16	12	6*D*_2_	3*D*_2_	C60	3
RM3	16	12	6*D*_2_	3*D*_2_	C40	3
RM4	14	10	6*D*_2_	3*D*_2_	C60	3
RM5	18	14	6*D*_2_	3*D*_2_	C60	3
RM6	16	12	7*D*_2_	2*D*_2_	C60	3
RM7	16	12	7*D*_2_	2*D*_2_	C85	3
RM8	16	12	7*D*_2_	2*D*_2_	C40	3
RM9	16	12	7*D*_2_	3*D*_2_	C60	3
RM10	14	10	7*D*_2_	3*D*_2_	C60	3
RM11	18	14	7*D*_2_	3*D*_2_	C60	3
RM12	16	12	8*D*_2_	3*D*_2_	C40	3
RM13	16	12	8*D*_2_	3*D*_2_	C60	3
RM14	18	14	8*D*_2_	3*D*_2_	C60	3
RM15	16	12	9*D*_2_	3*D*_2_	C60	3

**Table 2:** Material properties of grouting sleeves.

Type	Yield Strength (MPa)	Tensile Strength (MPa)	Elongation Ratio (%)
Ductile iron	≥370	≥600	≥3
Grade 45 steel	≥355	≥600	≥16
Q345	≥345	≥470	≥20

**Table 3:** Measured average mechanical properties of the steel bars.

Diameter (mm)	Yield Strength (MPa)	Tensile Strength (MPa)
12	435	650
16	436	654
18	427	632

**Table 4:** Measured mechanical properties of the grouting materials.

Strength of Reinforcement Material	Compressive Strength (MPa)	Average Value (MPa)
C40	51.1	51.8
	51.9	
	52.3	
C60	71.3	71.4
	72.5	
	70.4	
C85	92.7	94.3
	97.1	
	93.2	

**Table 5:** Characteristic values of the tensile test for the specimens.

No.	Ultimate Load (kN)	Tensile Strength (MPa)	$f_{mst}^{0}/f_{stk}$	Fracture Zone
S1-1	133.76	591.36	1.10	Weld heat-affected zone
S1-2	136.15	601.92	1.11	
S1-3	133.68	591.01	1.09	
S2-1	136.83	604.93	1.12	Predetermined failure zone
S2-2	136.71	604.40	1.12	
S2-3	136.23	602.28	1.12	
S3-1	137.23	606.70	1.12	
S3-2	136.97	605.55	1.12	
S3-3	136.47	603.34	1.12	

Note: $f_{mst}^{0}$ represents the measured tensile strength of the specimen, while $f_{stk}$ represents the standard value for the ultimate tensile strength of the steel reinforcement. The *f_stk_* value of the HRB400 grade steel bar is 540 MPa.

**Table 6:** Testing results of the novel reinforcement method of grouting sleeve connection.

No.	Ultimate Load (kN)	Tensile Strength (MPa)	$f_{mst}^{0}/f_{stk}$	Failure Type
RM1-1	122.13	607.43	1.12	P
RM1-2	122.56	609.57	1.13	P
RM1-3	121.56	604.60	1.12	P
RM2-1	121.12	602.41	1.12	P
RM2-2	123.11	612.30	1.13	P
RM2-3	122.32	608.38	1.13	P
RM3-1	127.34	633.22	1.17	P
RM3-2	92.86	-	0.86	M
RM3-3	84.13	-	0.77	N
RM4-1	90.32	586.72	1.09	P
RM4-2	98.18	637.78	1.18	P
RM4-3	95.69	621.61	1.15	P
RM5-1	165.52	650.45	1.20	P
RM5-2	161.48	634.57	1.18	P
RM5-3	160.06	628.99	1.16	P
RM6-1	123.45	614.00	1.14	P
RM6-2	122.76	610.56	1.13	P
RM6-3	124.16	617.53	1.14	P
RM7-1	123.01	611.81	1.13	P
RM7-2	122.76	610.56	1.13	P
RM7-3	121.98	606.68	1.12	P
RM8-1	123.11	612.30	1.13	P
RM8-2	122.67	610.12	1.13	P
RM8-3	122.78	610.66	1.13	P
RM9-1	130.58	649.33	1.20	P
RM9-2	130.28	647.84	1.20	P
RM9-3	122.64	609.85	1.13	P
RM10-1	95.93	623.16	1.15	P
RM10-2	95.68	621.54	1.15	P
RM10-3	91.11	591.85	1.10	P
RM11-1	161.93	636.34	1.18	P
RM11-2	162.60	638.98	1.18	P
RM11-3	162.03	636.74	1.18	P
RM12-1	124.12	617.33	1.14	P
RM12-2	123.11	612.30	1.13	P
RM12-3	122.71	610.32	1.13	P
RM13-1	127.66	634.81	1.18	P
RM13-2	130.18	647.34	1.20	P
RM13-3	131.02	651.52	1.21	P
RM14-1	161.98	636.54	1.18	P
RM14-2	161.12	633.16	1.17	P
RM14-3	164.96	648.25	1.20	P
RM15-1	90.13	-	0.83	M
RM15-2	130.07	646.79	1.20	P
RM15-3	130.75	650.17	1.20	P

**Table 7:** Parameters of the supplementary specimens.

Group No.	Diameter of Prefabricated Steel Bars *D*_1_ (mm)	Diameter of Post-Retrofitted Connecting Steel Bars *D*_2_ (mm)	Single-Sided Welding Length *L* (mm)	Protective Layer Thickness *D* (mm)	Strength of Reinforcement Material	Number of Specimens
S-RM1	16	12	4*D*_2_	2*D*_2_	C85	3
S-RM2	16	12	4*D*_2_	2*D*_2_	C60	3
S-RM3	16	12	4*D*_2_	3*D*_2_	C85	3
S-RM4	14	10	4*D*_2_	3*D*_2_	C60	3
S-RM5	18	14	5*D*_2_	2*D*_2_	C60	3
S-RM6	16	12	5*D*_2_	2*D*_2_	C85	3
S-RM7	16	12	5*D*_2_	3*D*_2_	C85	3
S-RM8	16	12	5*D*_2_	3*D*_2_	C60	3
S-RM9	16	12	4*D*_2_	No	No	3

**Table 8:** Testing results of the supplementary specimens.

No.	Ultimate Load (kN)	Tensile Strength (MPa)	$f_{mst}^{0}/f_{stk}$	Failure Type
S-RM1-1	121.75	605.54	1.12	P
S-RM1-2	121.66	605.09	1.12	P
S-RM1-3	120.93	601.46	1.11	P
S-RM2-1	120.63	599.97	1.11	P
S-RM2-2	123.67	615.09	1.14	P
S-RM2-3	121.87	606.14	1.12	P
S-RM3-1	121.93	606.44	1.12	P
S-RM3-2	121.57	604.65	1.12	P
S-RM3-3	121.11	602.36	1.12	P
S-RM4-1	92.37	600.32	1.11	P
S-RM4-2	92.39	600.47	1.11	P
S-RM4-3	94.28	612.75	1.13	P
S-RM5-1	152.91	601.21	1.11	P
S-RM5-2	154.51	607.48	1.11	P
S-RM5-3	154.35	606.88	1.12	P
S-RM6-1	121.45	604.05	1.12	P
S-RM6-2	121.04	602.01	1.11	P
S-RM6-3	121.48	604.20	1.12	P
S-RM7-1	120.64	600.02	1.11	P
S-RM7-2	121.13	602.46	1.12	P
S-RM7-3	121.27	603.15	1.12	P
S-RM8-1	121.87	606.14	1.12	P
S-RM8-2	121.84	606.00	1.12	P
S-RM8-3	121.46	604.10	1.12	P
S-RM9-1	66.12	-	0.61	N
S-RM9-2	44.77	-	0.41	N
S-RM9-3	50.12	-	0.46	N

Note: $f_{mst}^{0}$ represents the measured tensile strength of the specimen, while $f_{stk}$ represents the standard value for the ultimate tensile strength of the steel reinforcement. P refers to fracture failure of the prefabricated steel bar, and N refers to weld failure between the grouting sleeve and the post-retrofitted connecting steel bars.

## Data Availability

The raw data supporting the conclusions of this article will be made available by the authors on request.
